# 2,5-Dioxopyrrolidin-1-yl 2-methyl­prop-2-enoate

**DOI:** 10.1107/S1600536814005170

**Published:** 2014-03-15

**Authors:** Wayne H. Pearson, Shirley Lin, Lyle Isaacs

**Affiliations:** a Chemistry Department, United States Naval Academy, 572M Holloway Road, Annapolis, Maryland 21401, USA; b Department of Chemistry and Biochemistry, University of Maryland, College Park, Maryland 20742, USA

## Abstract

In the title compound, C_8_H_9_NO_4_, the pyrrolidine ring (r.m.s. deviation 0.014 Å) is almost normal to the mean plane of the propenoate group (r.m.s deviation 0.028 Å), making a dihedral angle of 86.58 (4)°. In the crystal, mol­ecules are linked *via* pairs of weak C—H⋯O hydrogen bonds, forming inversion dimers which stack along the c axis.

## Related literature   

For synthetic procedures, see: Batz *et al.* (1972 ▶); Rathfon & Tew (2008 ▶). For free radical polymerization and controlled free radical (ATRP) polymerizations to form homo- and copolymers, see: Batz *et al.* (1972 ▶); Rathfon & Tew (2008 ▶). For a background on post-polymerization modification to create functional polymers, see: Gauthier *et al.* (2009 ▶). For a review of topochemical polymerization in crystals, see: Matsumoto (2003 ▶). For a disscussion addressing the conformation of methyl substituents on alkenes, see: Deslongchamps & Deslongchamps (2011 ▶).
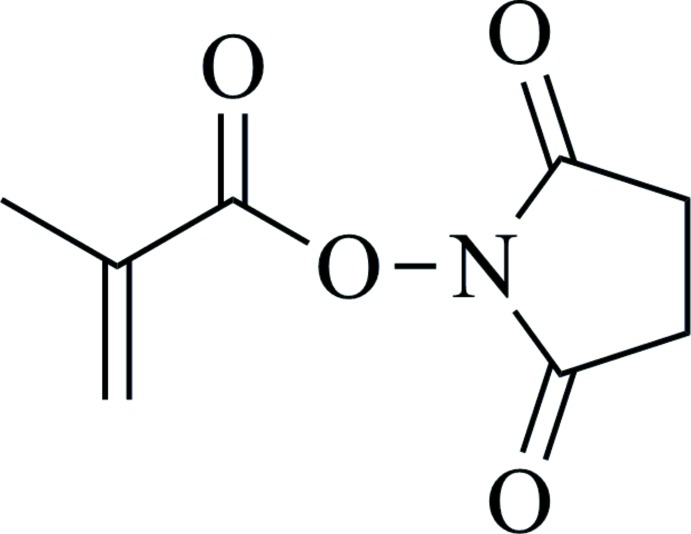


## Experimental   

### 

#### Crystal data  

C_8_H_9_NO_4_
*M* *_r_* = 183.16
Monoclinic, 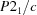

*a* = 9.6137 (8) Å
*b* = 10.9317 (9) Å
*c* = 8.4911 (7) Å
β = 102.522 (2)°
*V* = 871.14 (12) Å^3^
*Z* = 4
Mo *K*α radiation
μ = 0.11 mm^−1^
*T* = 173 K
0.24 × 0.14 × 0.07 mm

#### Data collection  

Bruker Kappa APEXII DUO diffractometer
Absorption correction: multi-scan (*SADABS*; Sheldrick, 2004 ▶) *T*_min_ = 0.884, *T*_max_ = 1.000
20817 measured reflections
1595 independent reflections
1353 reflections with *I* > 2σ(*I*)
*R*_int_ = 0.042

#### Refinement  

*R*[*F* ^2^ > 2σ(*F* ^2^)] = 0.036
*wR*(*F* ^2^) = 0.085
*S* = 1.06
1595 reflections
119 parameters
H-atom parameters constrained
Δρ_max_ = 0.19 e Å^−3^
Δρ_min_ = −0.17 e Å^−3^

### 

Data collection: *APEX2* (Bruker, 2013 ▶); cell refinement: *SAINT* (Bruker, 2013 ▶); data reduction: *SAINT*; program(s) used to solve structure: *SHELXS97* (Sheldrick, 2008 ▶); program(s) used to refine structure: *SHELXL97* (Sheldrick, 2008 ▶); molecular graphics: *ORTEP-3 for Windows* (Farrugia, 2012 ▶); software used to prepare material for publication: *SHELXL97* (Sheldrick, 2008 ▶).

## Supplementary Material

Crystal structure: contains datablock(s) I. DOI: 10.1107/S1600536814005170/zq2217sup1.cif

Structure factors: contains datablock(s) I. DOI: 10.1107/S1600536814005170/zq2217Isup2.hkl

Supporting information file. DOI: 10.1107/S1600536814005170/zq2217Isup3.cdx

Supporting information file. DOI: 10.1107/S1600536814005170/zq2217Isup4.cml

CCDC reference: 990423

Additional supporting information:  crystallographic information; 3D view; checkCIF report

## Figures and Tables

**Table 1:** Hydrogen-bond geometry (Å, °)

*D*—H⋯*A*	*D*—H	H⋯*A*	*D*⋯*A*	*D*—H⋯*A*
C7—H7*A*⋯O2^i^	0.98	2.54	3.393 (2)	145

Symmetry code: (i) 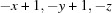.

**Fig. 1.**
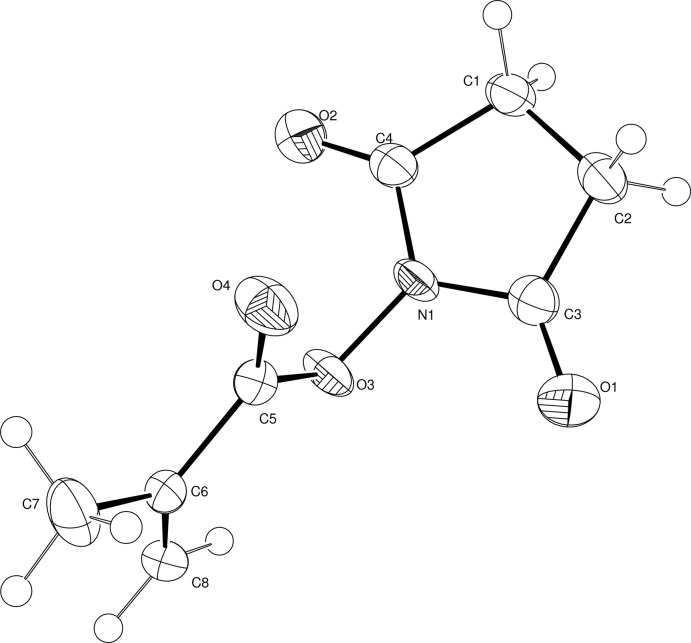
The molecular structure of the title compound, with atom labels and 50% probability displacement ellipsoids for non-H atoms

**Fig. 2.**
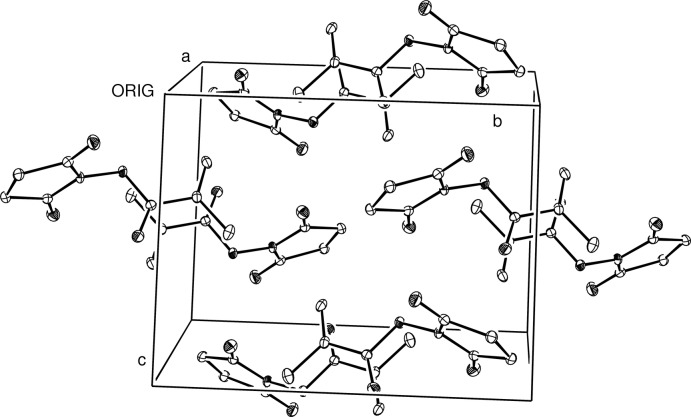
Unit cell view along the +a axis showing the lack of proper stacking for polymerization to occur.

## supplementary crystallographic information

### 1. Comment

The title compound is a monomer for free radical polymerization (Batz *et al.*, 1972) and controlled free radical (ATRP) polymerizations (Rathfon & Tew, 2008) to form homo- and copolymers. After preliminary polymerization, these polymers serve as candidates to undergo post-polymerization modification to create functional polymers (Gauthier *et al.*, 2009). A structure determination was undertaken to investigate the possibility of free radical, topochemical polymerization of this monomer while being exposed to X-ray radiation (Matsumoto, 2003). The molecular unit is shown in Figure 1. The crystal structure reveals that no polymerization has taken place. The asymmetric unit consists of a single monomer unit packed into a monoclinic cell with a volume of 871 Å^3^. While analysis of the intermolecular contacts within the unit cell reveals a close contact of 3.487 Å between the carbons of adjacent double bonds (C6 and C8), this contact occurs between a pair of adjacent molecules but is not maintained with additional molecules in order to achieve a favorable pathway for polymerization. Figure 2 shows the packing in the unit cell. The molecule is composed of two planar regions. Least-squares planar analysis reveals r.m.s. deviation from planarity for the pyrrolidine ring of 0.014 Å and 0.028 Å for the propenoate portion. The two planes are essentially normal to each other with an angle of 86.58 (4) degrees between least-squares planes. The conformation of the methyl H atoms is found to be *syn* to the vinylic proton. This is the preferred configuration by approximately 2 kcal/mol (Deslongchamps & Deslongchamps, 2011).

### 2. Experimental

Crystals of the title compound, C_8_H_9_NO_4_, were grown unintentionally from slow evaporation of a solution of the compound in 1:4 ethyl acetate:hexanes at 0 °C.

### 3. Refinement

Although all of the H-atoms were located in difference maps, H-atoms were placed at idealized positions and refined with a riding model having *U*_iso_(H) = 1.2 times *U*_eq_(C).

### Crystal data

**Table:**

C_8_H_9_NO_4_	*F*(000) = 384
*M**_r_* = 183.16	*D*_x_ = 1.397 Mg m^−^^3^*D*_m_ = 1.337 (2) Mg m^−^^3^*D*_m_ measured by flotation
Monoclinic, *P*2_1_/*c*	Mo *K*α radiation, λ = 0.71073 Å
*a* = 9.6137 (8) Å	Cell parameters from 8309 reflections
*b* = 10.9317 (9) Å	θ = 2.2–25.3°
*c* = 8.4911 (7) Å	µ = 0.11 mm^−^^1^
β = 102.522 (2)°	*T* = 173 K
*V* = 871.14 (12) Å^3^	Parallelpiped, colourless
*Z* = 4	0.24 × 0.14 × 0.07 mm

### Data collection

**Table:**

Bruker Kappa APEXII DUO diffractometer	1595 independent reflections
Radiation source: a micro-focus source with X-ray optics for beam focussing and collimation	1353 reflections with *I* > 2σ(*I*)
Graphite monochromator	*R*_int_ = 0.042
Detector resolution: 512 pixels mm^-1^	θ_max_ = 25.3°, θ_min_ = 2.2°
combination of ω and phi scans	*h* = −11→11
Absorption correction: multi-scan (*SADABS*; Sheldrick, 2004)	*k* = −13→13
*T*_min_ = 0.884, *T*_max_ = 1.000	*l* = −10→10
20817 measured reflections	

### Refinement

**Table:**

Refinement on *F*^2^	Primary atom site location: structure-invariant direct methods
Least-squares matrix: full	Secondary atom site location: difference Fourier map
*R*[*F*^2^ > 2σ(*F*^2^)] = 0.036	Hydrogen site location: inferred from neighbouring sites
*wR*(*F*^2^) = 0.085	H-atom parameters constrained
*S* = 1.06	*w* = 1/[σ^2^(*F*_o_^2^) + (0.0278*P*)^2^ + 0.472*P*] where *P* = (*F*_o_^2^ + 2*F*_c_^2^)/3
1595 reflections	(Δ/σ)_max_ < 0.001
119 parameters	Δρ_max_ = 0.19 e Å^−^^3^
0 restraints	Δρ_min_ = −0.17 e Å^−^^3^

### Special details

**Table:**

Geometry. All e.s.d.'s (except the e.s.d. in the dihedral angle between two l.s. planes) are estimated using the full covariance matrix. The cell e.s.d.'s are taken into account individually in the estimation of e.s.d.'s in distances, angles and torsion angles; correlations between e.s.d.'s in cell parameters are only used when they are defined by crystal symmetry. An approximate (isotropic) treatment of cell e.s.d.'s is used for estimating e.s.d.'s involving l.s. planes.
Refinement. Refinement of *F*^2^ against ALL reflections. The weighted *R*-factor *wR* and goodness of fit *S* are based on *F*^2^, conventional *R*-factors *R* are based on *F*, with *F* set to zero for negative *F*^2^. The threshold expression of *F*^2^ > σ(*F*^2^) is used only for calculating *R*-factors(gt) *etc*. and is not relevant to the choice of reflections for refinement. *R*-factors based on *F*^2^ are statistically about twice as large as those based on *F*, and *R*- factors based on ALL data will be even larger.CheckCIF detected one Alert level C stating that a large K value of 2.279 was detected in the Analysis of Variance. Examination of the *SHELX* output does reveal one large K value (1.967) for the Fc/Fc(max of 0.000). Examination of the K values as a function of resolution shows no large K values from inf to 0.83 Å. Our conclusion is that the large K value results from very weak relections in the 0.80 - 0.60 A region and should have a neglibile effect upon the final structural results while the inclusion of the data would minimize termination effects in the calculation of electron density.

### Fractional atomic coordinates and isotropic or equivalent isotropic displacement parameters (Å^2^)

**Table:**

	*x*	*y*	*z*	*U*_iso_*/*U*_eq_	
C1	0.82074 (17)	0.05447 (15)	0.09507 (19)	0.0323 (4)	
H1A	0.8376	0.0287	−0.0110	0.039*	
H1B	0.7895	−0.0176	0.1490	0.039*	
C2	0.95609 (18)	0.10915 (15)	0.19895 (19)	0.0326 (4)	
H2A	0.9894	0.0594	0.2973	0.039*	
H2B	1.0329	0.1126	0.1380	0.039*	
C3	0.91646 (17)	0.23566 (15)	0.24251 (18)	0.0301 (4)	
C4	0.71008 (17)	0.15357 (14)	0.07441 (18)	0.0283 (4)	
C5	0.72829 (16)	0.44523 (14)	0.06764 (17)	0.0261 (3)	
C6	0.66346 (16)	0.56484 (14)	0.09024 (18)	0.0270 (4)	
C7	0.6865 (2)	0.66054 (16)	−0.0276 (2)	0.0420 (4)	
H7A	0.6435	0.7377	−0.0037	0.063*	
H7B	0.6422	0.6342	−0.1373	0.063*	
H7C	0.7890	0.6723	−0.0188	0.063*	
C8	0.59118 (17)	0.58160 (15)	0.20439 (19)	0.0324 (4)	
H8A	0.5800	0.5160	0.2740	0.039*	
H8B	0.5504	0.6592	0.2168	0.039*	
N1	0.77548 (14)	0.25062 (11)	0.16597 (15)	0.0289 (3)	
O1	0.98688 (13)	0.31280 (11)	0.32386 (15)	0.0441 (3)	
O2	0.58963 (13)	0.15423 (11)	−0.00275 (15)	0.0408 (3)	
O3	0.70452 (12)	0.35922 (9)	0.17939 (13)	0.0319 (3)	
O4	0.79410 (13)	0.42090 (11)	−0.03174 (14)	0.0402 (3)	

### Atomic displacement parameters (Å^2^)

**Table:**

	*U*^11^	*U*^22^	*U*^33^	*U*^12^	*U*^13^	*U*^23^
C1	0.0440 (10)	0.0246 (8)	0.0283 (8)	0.0082 (7)	0.0076 (7)	−0.0019 (7)
C2	0.0377 (9)	0.0288 (9)	0.0328 (8)	0.0099 (7)	0.0108 (7)	0.0026 (7)
C3	0.0382 (9)	0.0269 (9)	0.0264 (8)	0.0032 (7)	0.0097 (7)	0.0037 (7)
C4	0.0385 (9)	0.0254 (8)	0.0234 (7)	0.0048 (7)	0.0118 (7)	0.0025 (6)
C5	0.0296 (8)	0.0246 (8)	0.0225 (7)	0.0025 (6)	0.0021 (6)	0.0002 (6)
C6	0.0273 (8)	0.0210 (8)	0.0281 (8)	0.0019 (6)	−0.0042 (6)	−0.0022 (6)
C7	0.0430 (10)	0.0295 (9)	0.0516 (11)	0.0068 (8)	0.0064 (8)	0.0112 (8)
C8	0.0356 (9)	0.0258 (8)	0.0321 (8)	0.0068 (7)	−0.0009 (7)	−0.0079 (7)
N1	0.0389 (8)	0.0183 (7)	0.0296 (7)	0.0104 (5)	0.0078 (6)	0.0001 (5)
O1	0.0487 (8)	0.0335 (7)	0.0470 (7)	−0.0019 (6)	0.0033 (6)	−0.0072 (6)
O2	0.0366 (7)	0.0415 (7)	0.0429 (7)	0.0065 (5)	0.0053 (6)	−0.0038 (6)
O3	0.0460 (7)	0.0209 (6)	0.0323 (6)	0.0125 (5)	0.0164 (5)	0.0030 (5)
O4	0.0551 (8)	0.0342 (7)	0.0370 (7)	0.0107 (6)	0.0226 (6)	0.0050 (5)

### Geometric parameters (Å, º)

**Table:**

C1—C4	1.502 (2)	C5—O4	1.1894 (18)
C1—C2	1.527 (2)	C5—O3	1.3895 (18)
C1—H1A	0.9900	C5—C6	1.479 (2)
C1—H1B	0.9900	C6—C8	1.322 (2)
C2—C3	1.502 (2)	C6—C7	1.497 (2)
C2—H2A	0.9900	C7—H7A	0.9800
C2—H2B	0.9900	C7—H7B	0.9800
C3—O1	1.202 (2)	C7—H7C	0.9800
C3—N1	1.380 (2)	C8—H8A	0.9500
C4—O2	1.2005 (19)	C8—H8B	0.9500
C4—N1	1.383 (2)	N1—O3	1.3862 (15)
			
C4—C1—C2	106.17 (13)	O4—C5—C6	126.43 (14)
C4—C1—H1A	110.5	O3—C5—C6	111.92 (12)
C2—C1—H1A	110.5	C8—C6—C5	121.40 (15)
C4—C1—H1B	110.5	C8—C6—C7	124.80 (15)
C2—C1—H1B	110.5	C5—C6—C7	113.80 (14)
H1A—C1—H1B	108.7	C6—C7—H7A	109.5
C3—C2—C1	105.84 (13)	C6—C7—H7B	109.5
C3—C2—H2A	110.6	H7A—C7—H7B	109.5
C1—C2—H2A	110.6	C6—C7—H7C	109.5
C3—C2—H2B	110.6	H7A—C7—H7C	109.5
C1—C2—H2B	110.6	H7B—C7—H7C	109.5
H2A—C2—H2B	108.7	C6—C8—H8A	120.0
O1—C3—N1	124.12 (15)	C6—C8—H8B	120.0
O1—C3—C2	130.27 (15)	H8A—C8—H8B	120.0
N1—C3—C2	105.60 (13)	C3—N1—C4	117.01 (13)
O2—C4—N1	124.70 (14)	C3—N1—O3	120.89 (13)
O2—C4—C1	130.03 (15)	C4—N1—O3	122.09 (13)
N1—C4—C1	105.28 (13)	N1—O3—C5	111.51 (11)
O4—C5—O3	121.65 (14)		
			
C4—C1—C2—C3	3.14 (16)	O1—C3—N1—O3	−0.2 (2)
C1—C2—C3—O1	179.04 (17)	C2—C3—N1—O3	−179.34 (12)
C1—C2—C3—N1	−1.86 (16)	O2—C4—N1—C3	−177.73 (15)
C2—C1—C4—O2	176.76 (16)	C1—C4—N1—C3	2.28 (18)
C2—C1—C4—N1	−3.24 (16)	O2—C4—N1—O3	1.3 (2)
O4—C5—C6—C8	179.43 (16)	C1—C4—N1—O3	−178.65 (12)
O3—C5—C6—C8	−0.8 (2)	C3—N1—O3—C5	84.50 (16)
O4—C5—C6—C7	0.1 (2)	C4—N1—O3—C5	−94.54 (16)
O3—C5—C6—C7	179.94 (13)	O4—C5—O3—N1	4.8 (2)
O1—C3—N1—C4	178.91 (15)	C6—C5—O3—N1	−175.03 (11)
C2—C3—N1—C4	−0.25 (18)		

### Hydrogen-bond geometry (Å, º)

**Table:**

*D*—H···*A*	*D*—H	H···*A*	*D*···*A*	*D*—H···*A*
C7—H7*A*···O2^i^	0.98	2.54	3.393 (2)	145

Symmetry code: (i) −*x*+1, −*y*+1, −*z*.
